# Melatonin alters the secondary metabolite profile of grape berry skin by promoting *VvMYB14*-mediated ethylene biosynthesis

**DOI:** 10.1038/s41438-021-00478-2

**Published:** 2021-03-01

**Authors:** Wanyun Ma, Lili Xu, Shiwei Gao, Xingning Lyu, Xiaolei Cao, Yuxin Yao

**Affiliations:** State Key Laboratory of Crop Biology, Collaborative Innovation Center of Fruit & Vegetable Quality and Efficient Production, College of Horticulture Science and Engineering, Shandong Agricultural University, Tai-An, Shandong 271018 China

**Keywords:** Secondary metabolism, Plant signalling

## Abstract

The interplay between melatonin and ethylene in the regulation of fruit metabolism and the underlying molecular mechanism of this interplay remain largely unclear. Here, widely targeted metabolomics analysis revealed a total of 464 metabolites present in berry skin. Among them, 27 significantly differentially accumulated metabolites (DAMs) were produced in response to melatonin treatment in the presence or absence of 1-MCP. Most of the DAMs were secondary metabolites, including flavonoids, phenolic acids, stilbenes, and flavonols. Additionally, the accumulation of 25 DAMs was regulated by melatonin via ethylene. RNA-seq analysis indicated that melatonin primarily regulated the pathways of plant hormone signal transduction and secondary metabolite biosynthesis via ethylene. Gene-metabolite association analysis showed that melatonin regulated the expression of the *VvSTS1*, *VvF3H*, *VvLAR2*, and *VvDFR* genes, suggesting that these genes may play key roles in regulating secondary metabolites in the skin; additionally, *VvMYB14* and *VvACS1* were suggested to be involved in the regulation of secondary metabolites. Further experiments revealed that melatonin induced the expression of *VvMYB14* and that VvMYB14 increased ethylene production by transcriptionally activating *VvACS1*, thereby affecting the accumulation of secondary metabolites. Collectively, melatonin promotes ethylene biosynthesis and alters secondary metabolite accumulation through the regulation of *VvACS1* by VvMYB14.

## Introduction

Grapevine is one of the most important fruit crop species worldwide, and the quality of grape berries mainly depends on their primary and secondary metabolites; moreover, the chemical composition of berries is primarily influenced by secondary metabolites, including alkaloids, terpenes, phenolics, and volatiles^1^. Grape berries are a rich source of natural antioxidant compounds (mainly polyphenols) that contribute to more than half of the global polyphenol market^2^. Grape berries are nonclimacteric fruits, and several signaling molecules, including melatonin and ethylene, participate in berry metabolism and ripening regulation^3^, although the mechanism underlying the ripening of nonclimacteric fruits, including grape berries, remains largely unclear.

The ethylene-mediated regulatory network of climacteric fruits is well known. In contrast, only a few studies have shown that nonclimacteric fruits, including those of grape, strawberry, and cherry, have a fully functional ethylene biosynthesis pathway. Peak ethylene production occurs during early development of grape berries and strawberry fruits;^4,5^ this pattern is typical for nonclimacteric fruits. Metabolomic analysis has shown that ethylene regulates a wide range of metabolites in nonclimacteric *Capsicum* fruits^6^. Additionally, the discovery of 1-methylcyclopropene (1-MCP) as a specific inhibitor of ethylene action has provided a powerful tool for elucidating ripening and senescence mechanisms in climacteric and nonclimacteric fruits and vegetables^7,8^. For example, both exogenous ethylene and 1-MCP alter ripening-related parameters, including anthocyanin accumulation, in grape^9^. Moreover, exogenous ethylene and 1-MCP promote and inhibit, respectively, the postharvest ripening of sweet cherry fruits^10^. Studies of nonclimacteric fruits challenged with 1-MCP have identified both ethylene-dependent and ethylene-independent ripening processes^7^.

Melatonin (N-acetyl-5-methoxytryptamine, MT) is a low-molecular-weight indole amine synthesized from L-tryptophan and functions as a pleiotropic molecule with diverse functions in plants^11^. It has been revealed that MT plays a role in regulating fruit ripening or postharvest senescence across various plant systems, such as those of grape, tomato, and banana^12–14^. In addition, MT has been reported to increase the content of phenols, anthocyanins, and flavonoids in grape berries^13^. Postharvest treatment with MT increases total phenols and anthocyanins in strawberry fruits^15^ and delays the loss of total anthocyanins, flavonoids, and phenols in litchi fruits^16^. Furthermore, MT increases the content of soluble sugars, particularly sucrose and sorbitol, in pear fruits^17^. Therefore, MT promotes fruit ripening and alters metabolite accumulation; however, the mechanism underlying these actions remains largely unknown. Several studies have shown that MT may function through interactions with other hormones, including ABA, ethylene, auxin, and cytokinins^18^. Our previous study indicated that MT treatment promotes grape berry ripening in part via other signaling molecules, such as ABA, H_2_O_2_ and, in particular, ethylene;^5^ additionally, MT increases the polyphenol content of berries via ethylene signaling^13^.

Additionally, several studies have demonstrated that MYB14 is involved in the regulation of metabolites. VvMYB14 is reported to transcriptionally regulate stilbene biosynthesis by specifically activating the promoters of the *STS* gene in grapevine^19^. Analysis of overexpression and mutant plants showed that *MtMYB14* is related to proanthocyanidin accumulation in the hairy roots and seeds of *Medicago truncatula*^20^. Moreover, ZmMYB14 functions as a key regulator of ZmBT1 and is closely related to the biosynthesis of starch by transcriptionally activating the expression of six starch synthesis-related genes in maize^21^. Although some target genes of MYB14 have been identified, the mechanism underlying the broad role of MYB14 in regulating metabolites needs further investigation. Additionally, in grape, *VvMYB14* was found to be significantly induced by MT in our previous study^13^, but it remains unclear whether VvMYB14 regulates secondary metabolism in response to MT.

To date, little is known about the global changes in metabolites caused by MT and about the interplay of MT and ethylene, particularly the underlying molecular mechanism. Therefore, the objective of the present study was to determine whether MT promotes ethylene biosynthesis and alters secondary metabolite accumulation through the regulation of *VvACS1* by VvMYB14. 1-MCP was applied to inhibit ethylene signaling in control and MT-treated berry skins. Widely targeted metabolomics and RNA-seq analysis were used to provide a comprehensive identification of metabolites and genes induced in response to melatonin in the presence or absence of 1-MCP. Transcriptional activation assays of *VvACS1* by VvMYB14 and overexpression and suppression of *VvMYB14* in grape calli were performed to demonstrate the role of *VvMYB14* in mediating the interaction of melatonin and ethylene.

## Results

### MT treatment of preveraison grape berries promotes ethylene production in the skin, which is not affected by 1-MCP

**Fig. 1:**
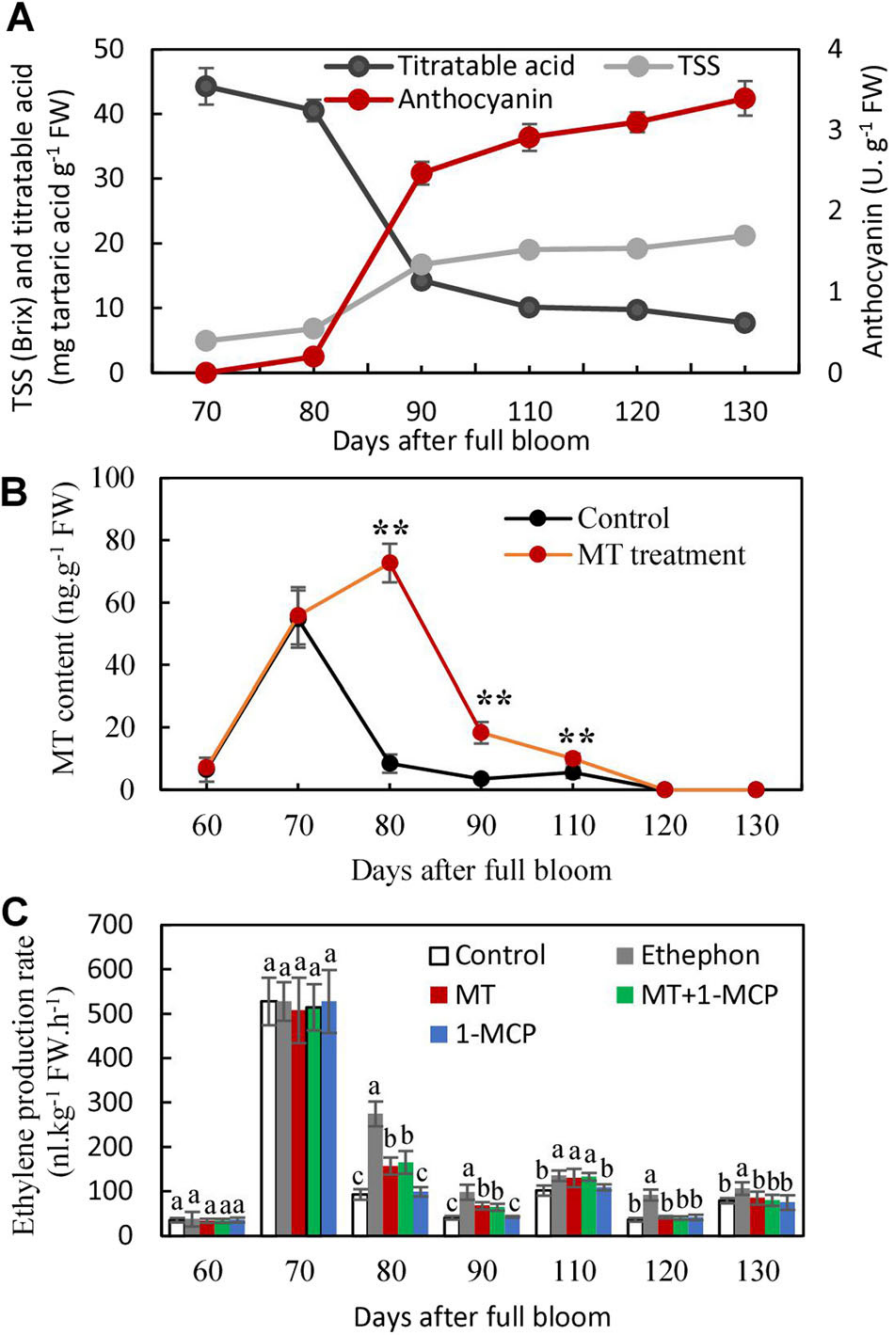
Changes in MT and ethylene production during ripening of control berry skins and those treated with MT, ethephon, MT + 1-MCP, or 1-MCP. Changes in the content of TSS, titratable acid, and anthocyanins were used to indicate the onset of ripening (**A**). The berries at 70 days after bloom were treated with 50 µM MT, 250 mg.l^−1^ ethephon, 50 µM MT plus 5 µl.l^−1^ 1-MCP or 5 µl.l^−1^ 1-MCP in panels B and C. FW fresh weight. The values represent the means ± SD of three replicates. *, Significant difference, *P* < 0.05; **, highly significant difference, *P* < 0.01. The values indicated by the same lowercase letters are not significant at *P* < 0.05 on the basis of Duncan’s multiple range test

TSS, titratable acid, and anthocyanin contents were measured to evaluate the occurrence of veraison (onset of berry ripening). Anthocyanins and TSS began to accumulate, and titratable acid began to decrease at 80 days after blooming (DAB) (Fig. 1A), indicating the occurrence of veraison at approximately this time point. The peaks of MT and ethylene production were detected at 70 DAB in the control berry skins. Afterward, the MT content and ethylene production rate declined sharply, and MT was undetectable past 120 DAB (Fig. 1B, C). MT treatment at 70 DAB largely increased MT levels in the berry skin and led to an 8.59-fold increase at 80 DAB compared with the level in the control skin (Fig. 1B). Additionally, MT and, in particular, ethephon, significantly increased ethylene production from 80 to 110 DAB and caused 47.12% and 194.99% increases in ethylene production, respectively, at 80 DAB (Fig. 1B). Therefore, melatonin and ethylene were primarily produced in preveraison berry skins, and MT treatment increased ethylene production. Additionally, 1-MCP was used to inhibit ethylene action, and its effects on ethylene production were determined in this study. Compared with the treatment of MT alone or the control, the application of 1-MCP in the presence or absence of MT did not exert significant effects on ethylene production (Fig. 1C). Therefore, the increases in ethylene production were attributed to MT in berry skins treated with MT + 1-MCP.

### Identification of differentially accumulated metabolites (DAMs) in response to MT and 1-MCP

**Fig. 2:**
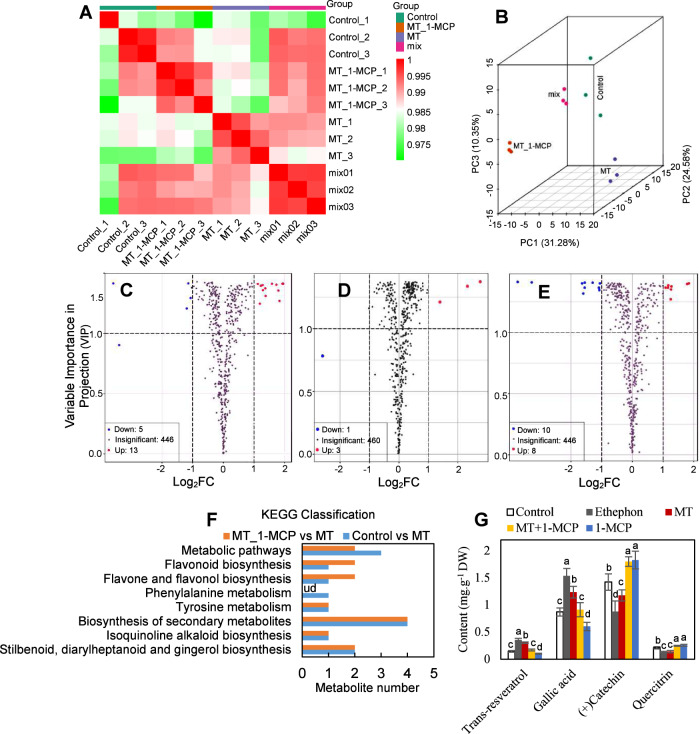
Identification and functional characterization of DAMs between control berry skins and those treated with MT (50 µM), ethephon (250 mg.l^−1^), 1-MCP (5 µl.l^−1^), or MT + 1-MCP. **A**, **B** Pearson’s correlation coefficients (**A**) and PCA (**B**) of the three samples and quality control sample (mix) for metabolomic analysis. **C**–**E** Volcano plot showing the DAMs from metabolomic analysis in the control vs MT (**C**), control vs MT_1-MCP (**D**), and MT_1-MCP vs MT (**E**) comparisons. **F** KEGG enrichment analysis of DAMs from metabolomic analysis in the MT_1-MCP vs MT and control vs MT comparisons. **G** Changes in the accumulation of the four metabolites in the control berry skins and those treated with ethephon, MT, MT + 1-MCP, or 1-MCP; the above metabolites were determined using berry skins collected at 110 DAB on an HPLC system. The control denotes berry skins treated with water. MT, ethephon, and 1-MCP denote berry skins treated with melatonin, ethephon, and 1-MCP, respectively. MT_1-MCP denotes berry skins treated with melatonin plus 1-MCP. ud undetectable

Berry skins collected at 110 DAB were used to detect changes in metabolite concentrations in response to MT and the ethylene receptor inhibitor 1-MCP using the widely targeted metabolomics approach. High correlation coefficients (Fig. 2A) and different clustering (Fig. 2B) of three biological replicates for each treatment indicated the strong reliability of the generated metabolomic data and the large effects of the MT and MT_1-MCP (treatment with MT plus 1-MCP) treatments on metabolites. After quality validation, a total of 464 metabolites were detected in the control berry skins and in those treated with MT and MT_1-MCP (Table S2).

A total of 27 DAMs between pairs of treatments were identified on the basis of a fold change ≥2 or ≤0.5 and a VIP ≥ 1. In total, 18 DAMs were identified between the control and MT groups, and only 4 DAMs were identified between the control and MT_1-MCP groups (Fig. 2C, D; Tables S3 and S4), indicating that inhibition of ethylene signaling by 1-MCP reduced the effects of MT on DAMs. Additionally, 18 DAMs were identified in the MT_1-MCP vs MT comparison (Fig. 2E; Table S5). KEGG enrichment analysis showed that the DAMs in the control vs MT and the MT_1-MCP vs MT comparisons had very similar KEGG classifications. These included biosynthesis of secondary metabolites; stilbenoid, diarylheptanoid, and gingerol biosynthesis; and biosynthesis of flavonoids, flavones, and flavonols (Fig. 2F).

Additionally, the contents of the four metabolites in berry skins under different treatments were measured to verify the role of MT in regulating DAMs via ethylene (Fig. 2G). Compared to the control, ethephon treatment largely increased the content of transresveratrol and gallic acid and decreased the content of catechin and quercitrin; in contrast, 1-MCP application led to contrasting results; therefore, ethylene played a role in regulating the accumulation of these four metabolites. MT treatment caused similar effects to those caused by ethephon, and the application of 1-MCP caused significant inhibitory effects on MT-induced changes in the content of the four metabolites. Therefore, MT regulated the accumulation of the four metabolites via ethylene.

Additionally, the 27 DAMs were normalized and classified into six groups based on their changes in abundance in the different samples (Table 1). The abundance of 14 DAMs in groups 5 and 11 DAMs in groups 1, 4, and 6 increased and decreased in response to melatonin, respectively. In contrast, the changes in DAMs caused by MT were alleviated and even reversed by 1-MCP. Therefore, the regulatory effects of melatonin on the 25 DAMs were mediated by ethylene, at least in part.

**Table 1:** Standardized intensity of DAMs in different samples

Class	Compounds	Control	MT	MT_1-MCP
Flavonoids	Delphinidin chloride, Luteolin 7-O-glucuronide, Quercitrin, Avicularin, Kaempferol, Quercetin 3,7-bis-O-β-D-glucoside			
Lipids	N-(2-hydroxyethyl)eicosapentaenoic acid			
Amino acid derivatives	S-methyl glutathione			
Amino acid derivatives	L-tyramine			
Others	Phenethylamine			
Phenolic acids	Piceid, Pallidol, 3-(4-Hydroxyphenyl)-propionic acid, Piceatannol, 3-Hydroxy-4-isopropylbenzylalcohol 3-glucoside-glucoside, Gallic acid			
Flavonoids	Propyl gallate			
Lipids	13-HOTrE(r)			
Organic acids	DL-P-hydroxyphenyllactic acid			
Stilbene	Resveratrol, Pterostilbene			
Others	α-Viniferin, 9,10-Dihydrophenanthrene, Digalloylglucose			
Flavonols	Pinocembrin, Catechin, Kaempferin			

### Identification of the changes in the transcriptome profile of berry skins in response to MT and 1-MCP

**Fig. 3:**
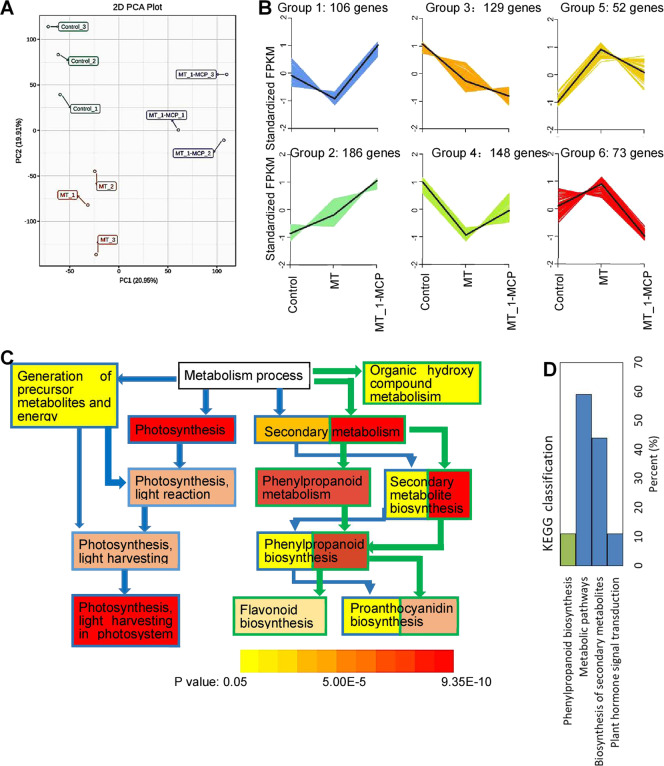
Expression patterns of DEGs in different samples and their enrichment based on GO and KEGG enrichment analyses. **A** PCA of the three skin samples based on the RPKM values. **B** Clusters of the DEGs according to their expression patterns in the three samples. **C** Genes whose expression significantly changed and that involved in metabolic processes according to GO enrichment analysis. The green and blue arrows and the shape outlines indicate significantly changed metabolic processes in the control vs MT and MT_1-MCP vs MT comparison groups, respectively. The background color of the shape represents the P value. **D** KEGG enrichment of the DEGs occurring simultaneously in the control vs MT and MT_1-MCP vs MT comparisons

RNA-seq analysis of the control skins and skins treated with MT and MT_1-MCP was conducted to quantify gene expression changes. High correlation coefficients (Fig. S1) and different clustering (Fig. 3A) of three biological replicates for each treatment indicated high reliability of the transcriptomic data. In total, the expression of 404 and 487 genes was significantly up- and downregulated, respectively, in the MT-treated skin compared with the control skin (Table S6). Furthermore, the expression of 410 and 655 genes was significantly up- and downregulated, respectively, in the MT-treated skin compared with the skins in the MT_1-MCP treatment (Table S7). The differentially expressed genes (DEGs) were classified into six groups according to the change patterns of the standardized FPKMs in the different samples (Fig. 3B; Table S8). Compared to the control, the MT treatment upregulated the expression levels of the genes in groups 5 and 6; however, the addition of 1-MCP reduced the extent of upregulated expression in group 5 and led to downregulated expression in group 6. In contrast, the opposite patterns were found for the genes in groups 1 and 4 under MT and 1-MCP treatment. Therefore, MT altered the expression of the above four groups of genes through ethylene to varying extents.

GO enrichment analysis showed that the DEGs in the control vs MT comparison group were primarily associated with secondary metabolism, including biosynthesis of phenylpropanoids, flavonoids, and proanthocyanidins, in terms of metabolic processes; in contrast, the DEGs in the MT_1-MCP vs MT comparison group were primarily related to photosynthesis and secondary metabolism (Fig. 3C). KEGG enrichment analysis indicated that the DEGs occurring simultaneously in the control vs MT and MT_1-MCP vs MT comparisons were primarily related to plant hormone signal transduction and biosynthesis of secondary metabolites, which included phenylpropanoid biosynthesis (Fig. 3D).

### Association analysis of DAMs and DEGs

**Fig. 4:**
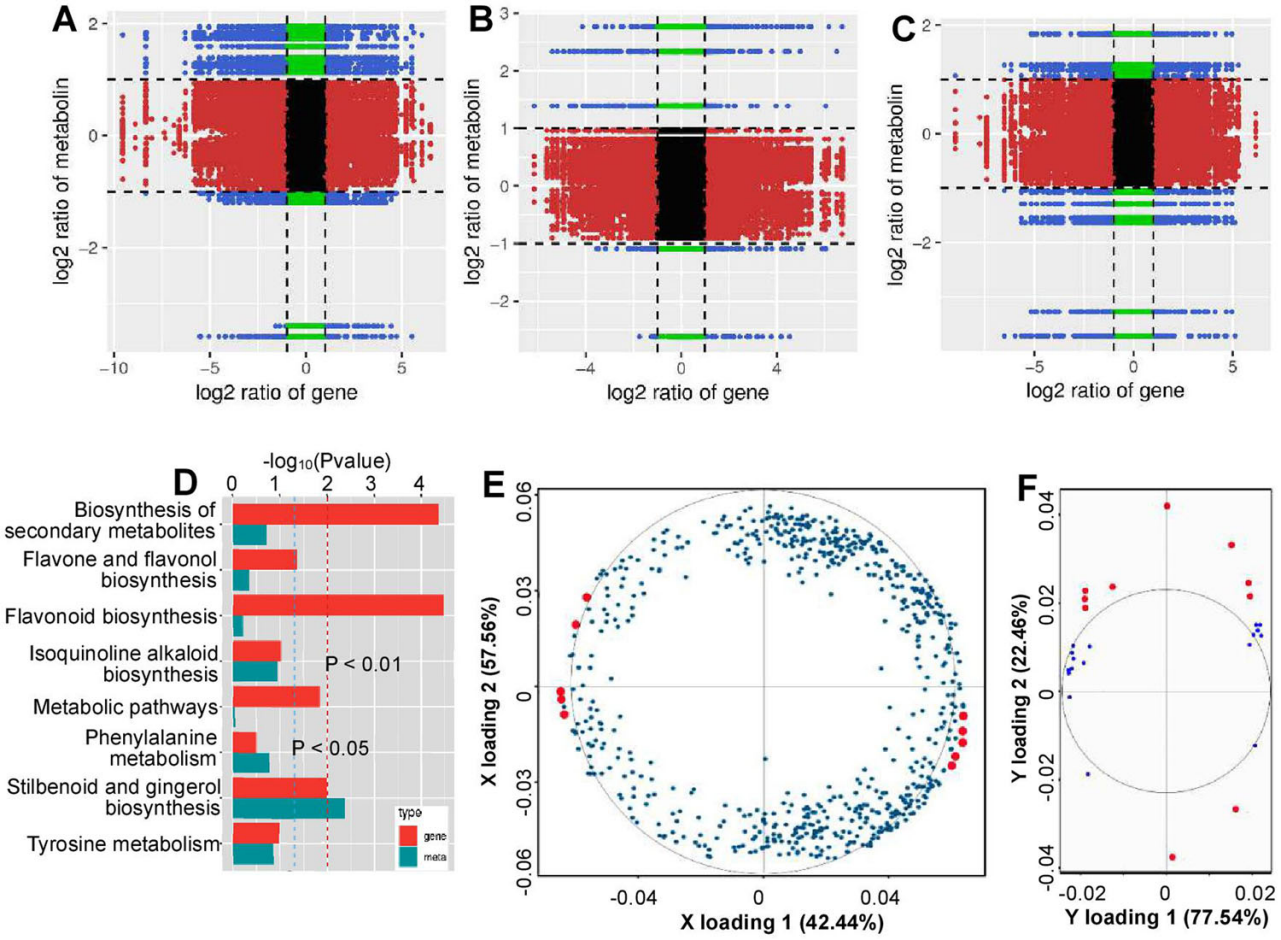
Transcriptomic and metabolomic variation and their associations between pairs of treatments. **A**–**C** Overview of transcriptomic and metabolomic variation in the control vs MT (**A**), control vs MT_1-MCP (**B**), and MT_1-MCP vs MT (**C**) comparisons. The *x*-axis represents the fold changes of gene expression, and the *y*-axis represents the fold change of the metabolites. Each point represents a gene/metabolite. The black dots represent unchanged genes/metabolites, the green dots represent DAMs with unchanged genes, the red dots represent DEGs with unchanged metabolites, and the blue dots represent both DEGs and DAMs. **D** KEGG enrichment of DEGs and DAMs that are correlated with each other. **E** O2PLS model correlated component loadings for the DEGs. **F** O2PLS model correlated component loadings for the DAMs. A longer distance of the dots representing DEGs (**E**) or DAMs (**F**) to the center dot of the circle means a stronger correlation with DAMs (**F**) or DEGs (**E**). The ten most influential DEGs and DAMs are highlighted with red loading plots in panels **E** and **F**, respectively

Nine quadrant diagrams were generated to systematically compare the variations in metabolites and their corresponding genes, with Pearson’s correlation coefficients >0.8. The DAMs and DEGs shown in quadrants 1 and 9 were negatively associated, while the DAMs and DEGs shown in quadrants 3 and 7 were positively associated (Fig. 4A–C). Compared with those in the control vs MT comparison, the DAMs and DEGs in the control vs MT_1-MCP comparison largely decreased (Fig. 4A, B), indicating the role of ethylene in mediating MT signaling. Additionally, the MT_1-MCP vs MT comparison showed that a total of 18 DAMs showed an association with DEGs, including 1191 genes in quadrants 3 and 7 and 1290 genes in quadrants 1 and 9 (Fig. 4C; Table S9). Moreover, stilbenoid, diarylheptanoid, and gingerol biosynthesis was the significantly changed KEGG pathway based on the DAMs and DEGs (Fig. 4D; Table S10). Further, to explore the relationship between the DAMs and DEGs, an O2PLS model was constructed, and the DEGs and DAMs were subsequently listed according to their distance to the center dot (Fig. 4E, F; Tables S11 and S12). In the X loading, *1-aminocyclopropane-1-carboxylate synthase 1* (*ACS1*), a key gene responsible for ethylene biosynthesis, was among the top 10 DEGs; additionally, three genes that encode transcription factors were in the top 50 DEGs: *VvMYB14*, *VvMYB86*, and *ethylene-responsive transcription factor RAP2-2*. *VvMYB14* and *VvACS1* were correlated with 17 and 15 DAMs (Table S11), respectively, suggesting their importance in regulating metabolites. In the Y loading, N-(2-hydroxyethyl) eicosapentaenoic acid, S-methyl glutathione, and pinocembrin were most strongly associated with the DEGs (Table S12).

**Fig. 5:**
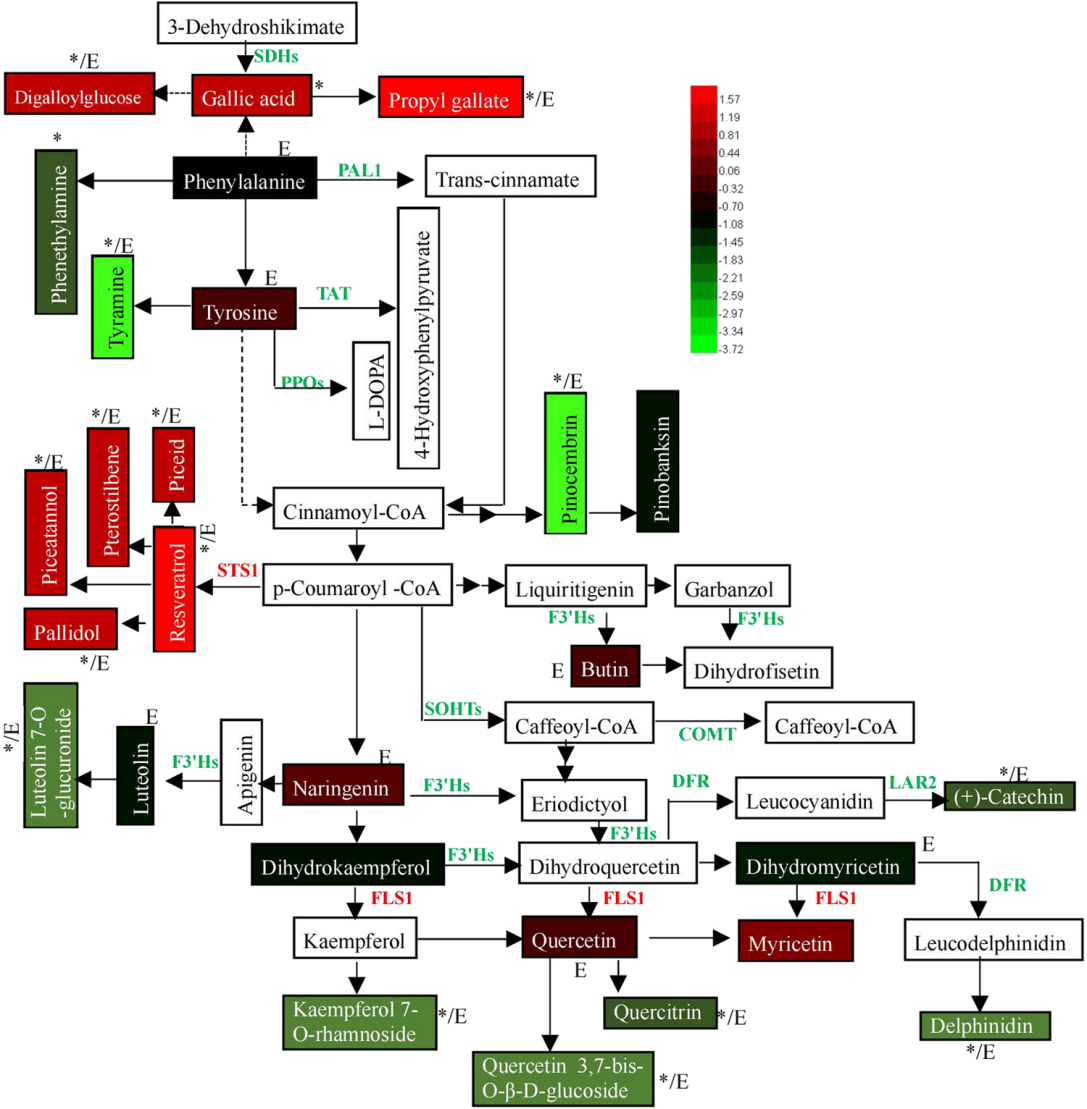
KEGG pathway analysis of the metabolites and genes that changed in response to melatonin and/or ethylene. The schematic was made by integrating the pathways of ko00945, ko00350, ko00360, ko00944, and ko00950. Each solid black arrow represents an enzyme-catalyzed reaction. The dotted line means that the uncertain enzyme-catalyzed processes are included. The boxes with white backgrounds indicate that metabolites were not detected in this study. The boxes with color represent the metabolites whose content changed in response to MT treatment, the difference in metabolite content is expressed as the log_2_(fold change) value, and the normalized values are shown on a color scale. The asterisk represents a significant difference, and E means that inhibition of ethylene signaling by 1-MCP reduced the effects of MT on DAMs. The genes whose expression was upregulated or downregulated are highlighted with red or green colors, respectively. PAL1 phenylalanine ammonia lyase, VIT_206s0004g02620, TAT tyrosine aminotransferase, VIT_212s0028g03200, PPO polyphenol oxidase, VIT_200s0480g00070 and VIT_210s0116g00560, FLS1 flavonol synthase, VIT_218s0001g03470, STS1 stilbene synthase, VIT_216s0100g00950, F3’H flavonoid-3’-hydroxylase, VIT_217s0000g07200 and VIT_217s0000g07210, SOHT shikimate O-hydroxycinnamoyltransferase, VIT_202s0087g00370 and VIT_209s0096g00660, COMT caffeoyl-CoA O-methyltransferase, VIT_212s0028g03110, DFR dihydroflavonol 4-reductase, VIT_218s0001g12800, LAR2 leucoanthocyanidin reductase, VIT_217s0000g04150, SDH shikimate dehydrogenase, VIT_214s0030g00660 and VIT_214s0030g00650

To better understand the changes in metabolites induced by MT via ethylene, a proposed metabolic pathway with annotations of variations in metabolites and candidate genes was presented (Fig. 5). The secondary metabolites whose content changed primarily originated from phenylalanine (a precursor of phenylpropanoid biosynthesis). The content of resveratrol and its four derivatives significantly increased in response to MT, and this increase was accompanied by the upregulation of *STS1* expression; additionally, these increases were mediated by ethylene. The content of gallic acid and its two derivatives also significantly increased, and the expression of two shikimate dehydrogenases (SDHs) involved in gallic acid biosynthesis was significantly downregulated. In contrast, the content of tyramine and eight other metabolites (excluding phenethylamine) significantly decreased, which was mediated by ethylene. The expression of key genes involved in flavonoid biosynthesis, including *F3H*s, *LAR2*, and *DFR*, was significantly downregulated, while that of *FLS1* was significantly upregulated.

### MT induces the expression of the VvMYB14 gene, whose product binds to the promoter of VvACS1 and activates its transcription

**Fig. 6:**
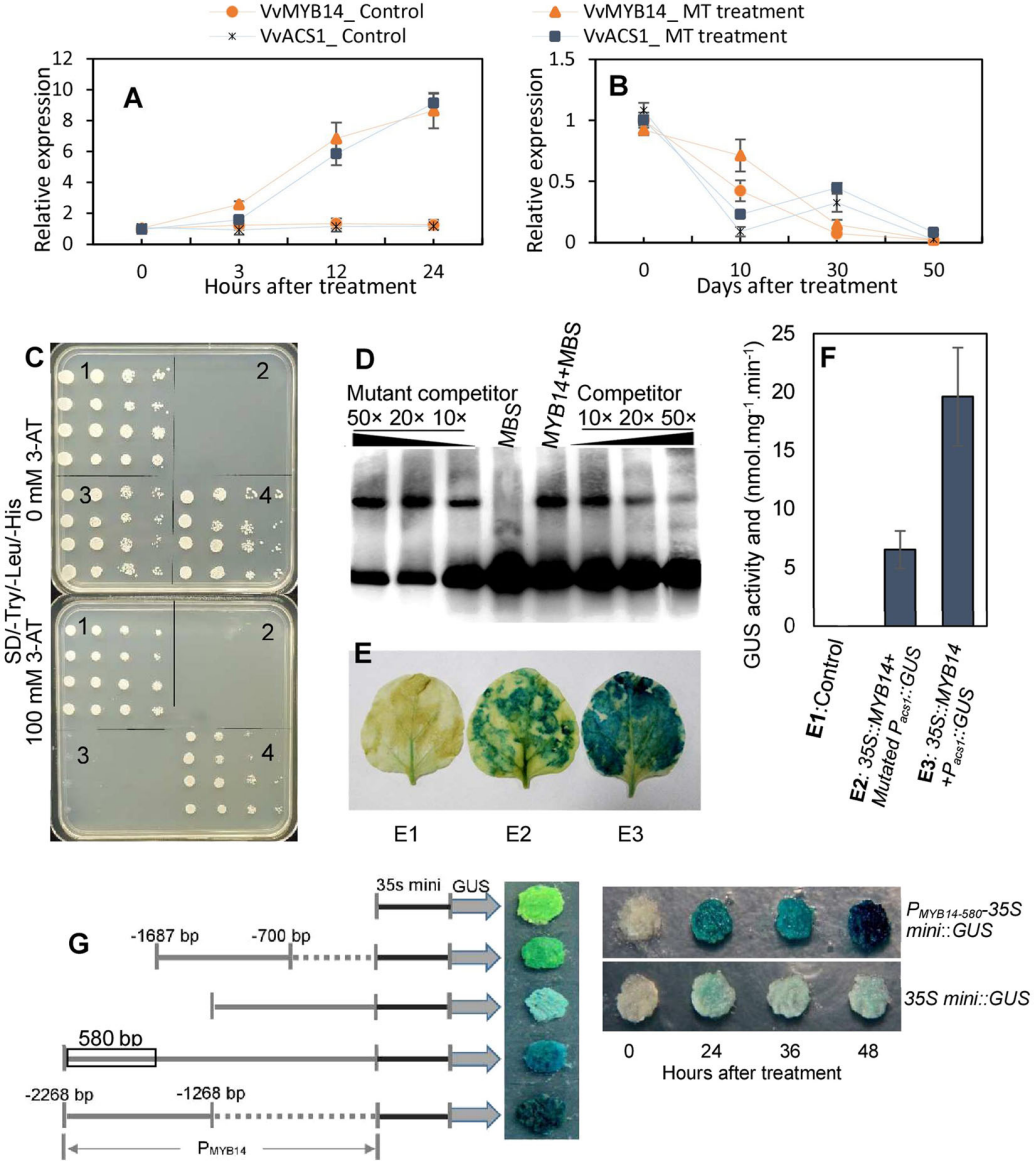
Expression of *VvACS1* and *VvMYB14* in response to MT and characterization of the transcriptional activation of *VvACS1* by VvMYB14. **A**, **B** MT treatment was applied at 70 DAB, and 50 days after treatment corresponds to the ripening stage of the grape berries in **B**. **C** Yeast one-hybrid assay. (1) P53-pHis2 + pGADT7-REC2-53, Rec-P53 and the P53 promoter, whose interactions have been confirmed, acted as positive controls; (2) pHis2 + pGADT7. (3) MBS-pHis2 + pGADT7. (4) MBS-pHis2 + pGADT7-MYB14. In panels 1–4, the yeast cells were diluted 1-, 10-, 100-, and 1000-fold, respectively, from left to right. 3-AT (3-amino-1,2,4-triazole) was used as a screening marker^54^. **D** Interaction of the VvMYB14-His protein with the labeled DNA probes for MBS elements or mutant MBS elements within the *VvACS1* promoter in an EMSA. **E**, **F** Histochemical staining (**E**) and GUS activity (**F**) analysis of the transactivation activity of VvMYB14 by binding to the *VvACS1* promoter. E1–E3 in panel **E** are annotated in panel **F**. The 1500 bp sequence upstream of the start codon was used as the *VvACS1* promoter, and the mutated *VvACS1* promoter refers to the *VvACS1* promoter sequence with the mutant MBS element that was the same as the mutant sequence used in the EMSA probe. **G** GUS staining of grape calli expressing the *VvACS1* promoter fragment-*35S* mini::GUS construct. The *VvACS1* promoter fragments are indicated by the gray solid lines, and the number denotes the length between the marked site and the start codon. P_MYB14-580_ represents the 580-bp fragment of the *VvMYB14* promoter, marked with a black box

In our previous study, *VvACS1* was proven to be a key gene controlling ACC biosynthesis and ethylene production^22^. Additionally, ethylene production (Fig. 1B) and the expression of *VvACS1* were induced by MT in berry skin (Fig. 6A, B). Therefore, *VvACS1* functions in controlling ethylene production in response to MT in berry skin. The presence of the MBS element in the promoter of *VvACS1* (Fig. S2) and expression association analysis (Fig. 6A, B; Fig. S3A) suggested the possible regulation of *VvACS1* transcription by VvMYB14 and VvMYB86. Further yeast one-hybrid (Y1H) assays excluded the possibility of interactions of VvMYB86 and the *VvACS1* promoter (Fig. S3B) and confirmed the binding of VvMYB14 to the MBS element within the *VvACS1* promoter (Fig. 6C).

To further confirm this interaction, an electrophoretic mobility shift assay (EMSA) was performed using the purified VvMYB14 protein and a biotin-labeled MBS element (Fig. 6D). VvMYB14 bound to the *VvACS1* promoter fragment containing MBS, and the binding was gradually reduced by the application of increasing amounts of unlabeled MBS competitor probe. In contrast, this competition was not detected when a mutated competitor was used. Therefore, the VvMYB14 protein specifically bound to the MBS element of the *VvACS1* promoter. Additionally, the regulation of the *VvACS1* promoter by VvMYB14 was determined using a β-glucuronidase (GUS) transactivation assay in tobacco leaves (Fig. 6E, F). Compared with tobacco leaves transformed with *35* *S*::*MYB14* and the mutant *Pacs1*::*GUS*, tobacco leaves cotransformed together with *35* *S*::*MYB14* and *Pacs1*::*GUS* constructs were more blue in color and showed higher GUS activity. Therefore, VvMYB14 increased *VvACS1* promoter activity.

Additionally, the expression of *VvMYB14* was strongly induced by MT (Fig. 6A, B). In particular, the transactivation ability of the *VvMYB14* promoter was enhanced by MT, and a 580-bp region was demonstrated to be the core region responding to MT (Fig. 6G). Collectively, we inferred that MT promoted ethylene production by inducing the expression of *VvMYB14* and therefore *VvACS1*.

### VvMYB14 mediates MT-induced ethylene production and modification of secondary metabolites in grape calli

**Fig. 7:**
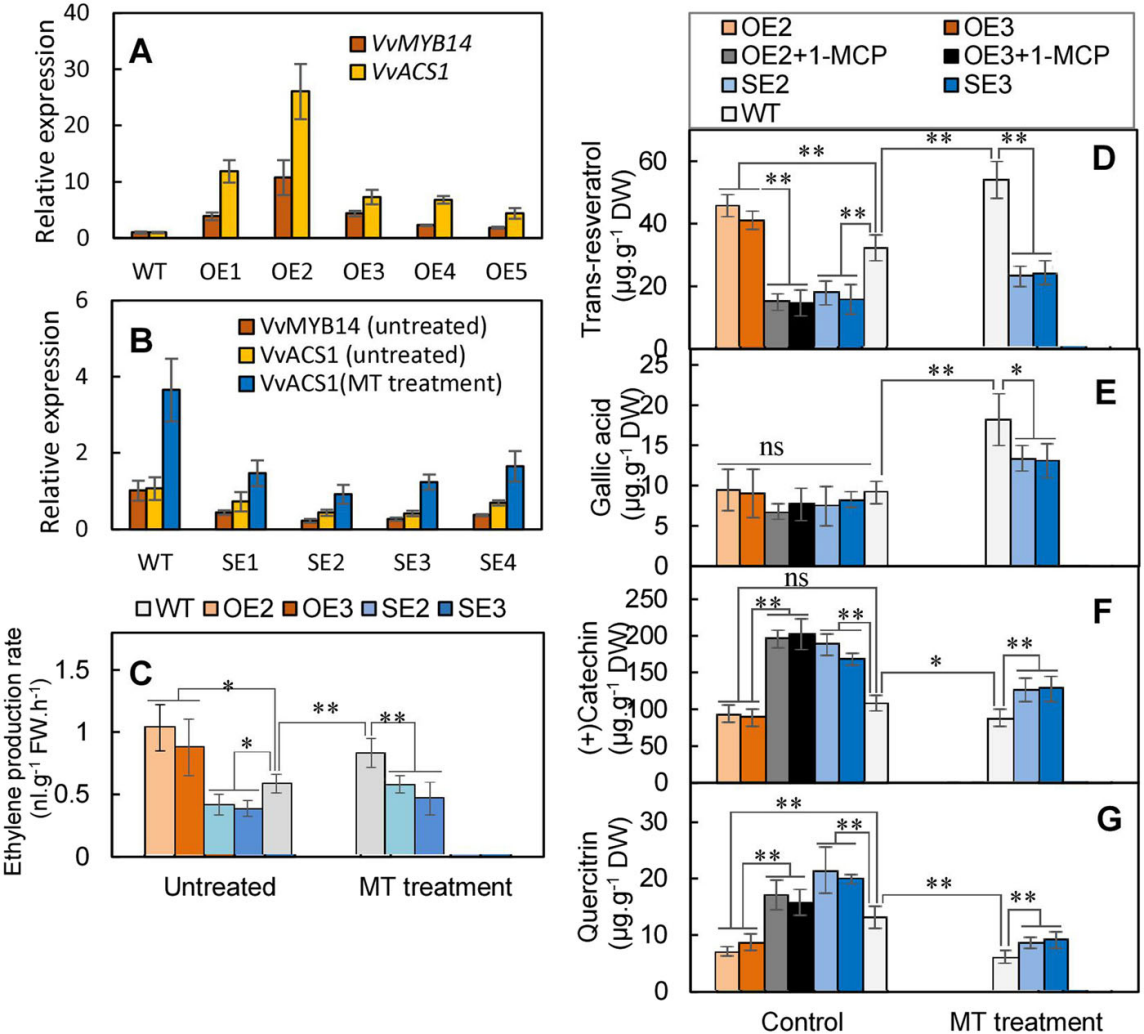
Changes in ethylene production and accumulation of the four metabolites in *VvMYB14*-overexpressing and VvMYB14-suppressed grape calli in the presence or absence of MT. **A**, **B** Expression levels of *VvMYB14* and *VvACS1* in *VvMYB14* overexpression lines (OEs) (A) and suppression lines (SEs) (B). **C**–**G** Ethylene production (**C**) and content of four metabolites (**D**–**G**) in the two overexpression lines and two suppression lines in the presence or absence of MT. The OEs were treated with 1-MCP to evaluate the effects of the inhibition of ethylene signaling on the contents of the four metabolites

To verify the above inference, transgenic grape calli with different levels of *VvMYB14* expression were obtained, including five overexpression lines (OE1-5) and four suppression lines (SE1-4). Overexpression of *VvMYB14* increased the expression levels of *VvACS1*, while suppression of *VvMYB14* decreased *VvACS1* expression (Fig. 7A, B); additionally, the MT-induced increase in *VvACS1* expression was largely reduced by VvMYB14 suppression (Fig. 7B). Therefore, MT enhanced *VvACS1* expression via *VvMYB14*.

Additionally, two overexpression lines (OE2 and OE3) and two suppression lines (SE2 and SE3) with the highest and lowest expression levels of *VvMYB14*, respectively, were used to determine the effects of the *VvMYB14* expression level on ethylene production and metabolite contents. *VvMYB14* overexpression significantly increased ethylene production, while its suppression led to the opposite results. MT increased ethylene production compared to that of the untreated control, and the MT-induced promotion of ethylene production was reduced in the suppression lines (Fig. 7C). Similar changes in transresveratrol content were found in the WT, overexpression lines and suppression lines; additionally, the transresveratrol content was largely reduced in response to 1-MCP application in the overexpression lines (Fig. 7D). The gallic acid content significantly increased in response to MT, but the changes in the *VvMYB14* expression level did not exert significant effects on the gallic acid content under the control conditions; however, *VvMYB14* suppression reduced the MT-induced increase in gallic acid (Fig. 7E). In contrast, melatonin decreased the content of catechin and quercitrin, but overexpression and suppression of *VvMYB14* decreased and increased their contents, respectively, in the presence or absence of MT treatment; additionally, the content of catechin and quercitrin largely increased in response to 1-MCP in the overexpression lines (Fig. 7F, G). Collectively, MT at least partially promoted ethylene production via VvMYB14 and therefore altered the accumulation of metabolites.

## Discussion

### MT may influence ethylene production in berries in a skin-specific manner

The MT concentration in the Merlot berry skins decreased with increasing berry ripening (Fig. 1B). However, opposite trends have been observed in grape seed and flesh, where MT concentrations increased during ripening^23^. Therefore, different accumulation patterns of MT occur in the skin and in other berry tissues. Additionally, the ethylene release peak has been shown to occur before veraison not only in Merlot berry skin (Fig. 1B) but also in whole berries of Moldova and Cabernet Sauvignon^5,24^. In contrast, the peak MT content in the berry skin occurred at preveraison, which was consistent with the ethylene release peak (Fig. 1C); however, the MT peak occurred more than one month later than the ethylene release peak did in Moldova berries^5^. Therefore, different interplay mechanisms of ethylene and MT might occur in berry skin and in other berry tissues.

Increasing amounts of evidence have shown that MT promotes ethylene biosynthesis in fruits. In this study, MT treatment promoted ethylene production in berry skins (Fig. 1B); similar results were also found in Moldova berries^5^. However, during tomato and banana postharvest ripening, MT treatment promotes and reduces ethylene production, respectively, through regulation of the expression of *ACO1* and *ACS1*^12,14^. Moreover, MT pretreatment decreased ethylene levels in alfalfa under waterlogging conditions^25^. A combination treatment of both MT and *Fusarium* wilt increased ethylene levels, whereas MT alone inhibited ethylene production in banana leaves^26^. The above opposite effects suggest that other regulators may mediate the interplay between MT and ethylene. This inference is supported by our previous study showing that MT promotes ethylene production in part via ABA during berry ripening^13^. Therefore, the effects of MT behavior on ethylene are complex and may largely depend on environmental factors, plant tissue and even developmental stage, and skin-specific interplay may occur in grape berries.

### Key secondary metabolic pathways modified by MT via ethylene in berry skins

1-MCP is an ethylene action inhibitor that binds to the cellular ethylene receptor and effectively inhibits ethylene responses^8^ and has been indicated to be a powerful tool for the inhibition of fruit ripening and senescence by blocking ethylene signaling in various nonclimacteric fruit crop species, including grape, cherry, citrus, and strawberry^27^. In addition to these major effects, 1-MCP has differing and sometimes contradictory effects on fruit ethylene production; e.g., 1-MCP has no significant effects on ethylene production in grape berries (Fig. 1C)^28^ but inhibits ethylene production in peach fruits^29^. In the present study, 1-MCP was also used to inhibit ethylene signaling and evaluate the role of MT in modifying secondary metabolic pathways via ethylene. Changes in DAMs (groups 4 and 5 in Table 1) and DEGs (groups 4 and 5 in panel B of Fig. 3) caused by MT were alleviated by 1-MCP, indicating that MT functioned at least in part via ethylene. In contrast, 1-MCP treatment reversed the changes in DAMs (groups 1 and 6 in Table 1) and DEGs (groups 1 and 6 in panel B of Fig. 3) caused by MT. This may have occurred because these DAMs and DEGs were highly dependent on ethylene, and their changes were primarily regulated by ethylene rather than MT, which was supported by the similar increases in catechin and quercitrin in berry skins treated with MT + 1-MCP and 1-MCP compared to the control (Fig. 2G). Notably, compared with the MT treatment, the MT + 1-MCP treatment produced additive effects on the DAMs (groups 2 and 3 in Table 1) and DEGs (groups 2 and 3 in panel B of Fig. 3), suggesting that these DAMs and DEGs may be antagonistically regulated by MT and ethylene and that the application of 1-MCP reduced the effects of ethylene, thereby improving the function of MT.

Resveratrol, gallic acid and their derivatives constituted the metabolites whose content significantly increased in response to MT via ethylene (Fig. 5). STS is the key enzyme responsible for the biosynthesis of resveratrol and its derivatives^30^, and the significant increase in resveratrol and its derivatives may be attributed to the upregulation of *STS1* expression (Fig. 5). Additionally, the MT-induced increases in the content of resveratrol and its derivatives and the expression level of *STS1* largely depend on ethylene (Fig. 5; Table S8), suggesting a role of ethylene in this process. In fact, ethylene has been reported to trigger the upregulation of stilbene biosynthesis-related gene expression and increase stilbene biosynthesis in grape and peanut^31,32^. In particular, the large decrease in resveratrol in the *VvMYB14*-suppressed calli and in the calli treated with 1-MCP demonstrated the role of ethylene in mediating the regulation of resveratrol by MT (Fig. 7C, D). Additionally, our recent study revealed that MT increased the expression of another *STS* gene by decreasing its promoter methylation^33^. Therefore, STSs may represent a key point in the pathway regarding the regulation of resveratrol by MT or MT via ethylene.

Although it has long been recognized that plants, bacteria, and fungi synthesize and accumulate gallic acid, the pathway leading to its synthesis is largely unknown. Gallic acid biosynthesis from 3-dehydroshikimate, an intermediate in the shikimate pathway, has been reported^34^, and two grape shikimate dehydrogenases (VvSDH3 and VvSDH4) have been shown to be involved in gallic acid biosynthesis in grapevine^35^. However, in the present study, the expression levels of *VvSDH3* and *VvSDH4* were significantly downregulated in response to MT, although the content of gallic acid and its derivatives significantly increased (Fig. 5). Therefore, the shikimate pathway may not be the key pathway for gallic acid biosynthesis in berry skin under MT treatment. Additional studies are needed to reveal the MT-induced increase in gallic acid and its derivatives.

As shown in Fig. 5, the pathways mediated by F3’H, DFR, and LAR2 may control the decreases in delphinidin, catechin, and luteolin 7-O-glucuronide contents under MT treatment. F3’H catalyzes the hydroxylation of the 3′ position of the B-ring of flavonoids, including naringenin and dihydrokaempferol. Mutation and downregulation of *F3’H* result in compositional changes in flavonols^36^ and in delphinidin accumulation^37^, respectively. The expression of *F3’H* and *F3'5’H* genes directly affects the accumulation of anthocyanin compounds in grape berry skin^38^. DFR shows a preference for dihydroquercetin and dihydromyricetin as substrates to produce anthocyanins in cultivars of *Vitis vinifera*^39^. Therefore, decreases in the expression of *F3’Hs* and *DFR* may reduce the synthesis of delphinidin, catechin, and luteolin and its derivatives by reducing the amount of their precursors. Additionally, LAR directly catalyzes the biosynthesis of catechin from leucocyanidin^40^, and the downregulation of *VvLAR2* expression directly contributes to a decrease in catechin. The synthesis of flavonol aglycones is catalyzed by FLS, which uses dihydroflavonols as substrates^41^. The increase in the expression of *VvFLS1* increased the contents of myricetin and quercetin, but they did not reach a significant level (Fig. 5). In contrast, the contents of kaempferol 7-O-rhamnoside, quercetin 3,7-bis-O-β-D-glucoside, and quercitrin significantly decreased, suggesting that other genes play a role. Here, the significant decrease in the content of the above compounds was dependent on ethylene, at least in part (Fig. 5; Tables S3–S5). However, various effects of ethylene on the content of secondary metabolites, including anthocyanins and catechin, have been reported^42,43^. Therefore, it is suggested that the regulation of secondary metabolites may be attributed to the combined effects of MT and ethylene in berry skin.

### VvMYB14 participates in the MT signaling pathway involved in the regulation of secondary metabolism

Gene expression and promoter assays revealed the responses of *VvMYB14* to MT (Fig. 6A, G). Additionally, the suppression of *VvMYB14* reduced the effects of MT on ethylene and the detected secondary metabolites (Fig. 7C–G). Therefore, VvMYB14 participates in the MT signaling pathway. MYB14 may function in two ways in the MT signaling pathway. First, MYB14 responds to MT and directly activates gene expression. In grape, VvMYB14 and VvMYB15 are known to be involved in the transcriptional regulation of *VvSTS* genes and to control stilbene levels in response to stresses^19^. MYB14 and MYB5 have been reported to regulate the proanthocyanidin pathway in seeds of *M. truncatula*, and their synergistic physical interactions increase the transcription of target proanthocyanidin pathway genes such as anthocyanidin reductase and anthocyanidin synthase^20,44^.

Second, MYB14 may serve as a bridge between MT and other signaling molecules, thereby affecting secondary metabolism via other signaling molecules. A large number of studies in different species have shown that ethylene regulates the accumulation of secondary metabolites. For example, ethylene regulates anthocyanin and proanthocyanidin biosynthesis via the ethylene response factor MdERF1B in apple^45^, ethylene modulates flavonol accumulation in *Arabidopsis*^46^, and ethylene regulates polyphenol metabolism in grape berries^47^. Here, we revealed that VvMYB14 largely regulated secondary metabolism via ethylene signaling (Fig. 7). Additionally, Myb14 overexpression impacts the JA-related transcriptome and stimulates terpene and anthocyanin accumulation^48,49^. The broad effects of MYB14 on gene expression and metabolites also suggest that MYB14 functions by regulating signaling pathways. For example, *LjMYB14*-overexpressing lotus plants show increased expression of genes involved in the general phenylpropanoid pathway and genes encoding enzymes of the isoflavonoid pathway^50^. Moreover, MYB14 has been identified as a putative regulator of a broad defense response involving flavonoids and isoprenoids in loblolly pine (*Pinus taeda*)^51^.

In summary, widely targeted metabolomics analysis revealed 27 DAMs whose abundance significantly changed in response to melatonin, of which the changes of 25 DAMs were mediated by ethylene. Transcriptome analyses indicated that melatonin primarily affects the pathways of plant hormone signal transduction and biosynthesis of secondary metabolites via ethylene. Association analysis of the DAMs and DEGs revealed that *F3’H*s, *STS*, *DFR*, and *LAR2* play key roles in regulating DAMs under melatonin treatment. Additionally, melatonin induced the expression of VvMYB14, which increased ethylene production by transcriptionally activating *VvACS1*, thereby altering the accumulation of secondary metabolites.

## Materials and methods

### Plant materials and growth conditions

The present experiment was conducted at an experimental vineyard in Tai-An city, Shandong Province, China. Each vine had 10 vertical fruiting shoots on the horizontal cordon, and each fruiting shoot was controlled to produce a cluster. Preveraison Merlot grape (*Vitis vinifera*) berries at 70 days after full bloom (DAB) were subjected to MT treatment and 1-MCP treatment. The grape clusters on the vine were completely soaked for 5 s in a solution of 50 µM MT + 0.05% Triton X-100, 50 µM melatonin + 5 µl l^−1^ 1-MCP + 0.05% Triton X-100 or 250 mg l^−1^ ethephon^5^. Treatment with 0.05% Triton X-100 via the same method was used as a control^5^. Each treatment included three replications, and each replication comprised 6 vines. Approximately 180 berries from the shoulder, middle, and tail of each cluster at different days after treatment were collected for subsequent experiments. In addition, discs of tissue cut from Merlot berry skin were cultured on MS media comprising 0.1 mg l^−1^ IBA and 1.5 mg l^−1^ TDZ to induce nonembryogenic callus development. The obtained calli were subcultured on MS media comprising 0.59 g l^−1^ 2-(N-morpholino)ethanesulfonic acid, 10 mg l^−1^ picloram, 2.2 mg l^−1^ thidiazuron, 0.8 g l^−1^ activated carbon, 30 g l^−1^ sugar, and 7 g l^−1^ agar at 25 °C under dark conditions.

### Determination of anthocyanin, total soluble solids (TSS), and titratable acid contents

The total anthocyanins in the berry skins were extracted and spectrophotometrically measured according to the methods described by Xu et al^5^. Fresh berry pulp was ground to a homogenate and filtered, and the filtrate was used for the determination of TSS and titratable acid contents. The TSS content was measured using a digital-display sugar meter (PAL-1; Atago, Tokyo, Japan), and the titratable acid content was determined by titration of the filtrate with 0.1 M NaOH to an endpoint, at pH 8.3.

### Determination of the MT content and ethylene production rate

MT was extracted and determined according to the methods of Xu et al^5^. The primary extraction procedures included preliminary extraction via an ultrasonic bath in methanol, evaporation of the extraction solution to dryness, and purification of the extract using a C_18_ solid-phase extraction cartridge (ProElut^TM^; Dikma, China). MT was determined using an UHPLC-MS system in conjunction with an ACQUITY UHPLC system and a QTOF micro–mass spectrometer (Waters, Milford, MA, USA). The parameters were as follows: mobile phase, 0.05% (v/v) acetic acid and methanol at 0.3 ml min^−1^; column temperature, 25 °C; capillary temperature, 300 °C; spray voltage, 3000 V; auxiliary pressure, 15 V; and sheath pressure, 35 V.

Five grams of berry skin was enclosed in a 100-mL jar and incubated for 3 h at 25 °C. Five milliliters of the headspace gas was then withdrawn from each jar using an air-tight syringe for ethylene determination. The ethylene concentration was determined using a GC-9A gas chromatograph (Shimadzu, Kyoto, Japan). The ethylene production rate was calculated on the basis of the ethylene concentration, incubation time, and skin weight^5^.

### Widely targeted metabolomics analysis

Metabolome extraction and analysis were performed by a commercial company (Metware Biotechnology Co., Ltd., Wuhan, China). In brief, the lyophilized berry skin was ground in a mixer mill (MM 400, Retsch) and then extracted with 70% methanol, followed by absorption (CNWBOND Carbon-GCB SPE Cartridge) and filtration (SCAA-104, 0.22 μm pore size) (ANPEL, Shanghai, China). The filtrate was analyzed using a UPLC-ESI-MS/MS system in conjunction with UPLC (Shim-pack UFLC Shimadzu CBM30A system) and MS (Applied Biosystems 4500 Q TRAP). The UPLC and MS conditions were set according to the methods described by Guo et al^52^. The metabolites were identified using the Metware database (MWDB). The metabolite abundances were quantified according to their peak areas. Metabolites were considered to have differentially accumulated when the variable importance in projection (VIP) was ≥1 and the absolute log_2_(fold change) was ≥1.

### Extraction and determination of the four metabolites

Transresveratrol, gallic acid, catechin, and quercitrin were extracted and determined according to the methods of Xu et al.^13^ and Sun et al.^53^. The primary extraction process included ultrasonication in a methanol solution, filtration, evaporation of the filtrate to dryness, and dissolving of the residue in chromatography-grade methanol. The determination of the metabolites was performed on an HPLC system (Waters 600, Waters, Milford, MA, USA). Resveratrol was isolated via gradient elution from 5% (V/V) acetonitrile to 75% acetonitrile and then measured at 307 nm. The other three phenolics were isolated as follows: 90% A (water:acetic acid, 98:2) and 10% B (acetonitrile) for 30 min, 65% A and 35% B for 42 min, and then 90% A and 10% B for 45 min. The signal was monitored at 280 nm.

### RNA-seq and quantitative RT–PCR

Sequencing libraries were constructed using a NEBNext^®^ Ultra^TM^ RNA Library Prep Kit for Illumina^®^ (#7530 L, NEB, USA) according to the manufacturer’s instructions. The libraries were sequenced on an Illumina HiSeq 4000 platform after a series of preparatory procedures, primarily those involving determinations of the RNA concentration and insert size and clustering of the index-coded samples. One hundred fifty-base pair paired-end reads were generated, and the clean reads were assembled into transcripts using Cufflinks, with the grape genome (http://genomes.cribi.unipd.it/grape/) used as a reference. Reads per fragment per kilobase of transcript per million mapped reads (RPKM) were used to quantify unigene expression levels, and the DEGs were screened in accordance with the following criteria: false discovery rate ˂0.05 and absolute log_2_(fold change) ≥1. Real-time quantitative PCR was performed using SYBR Green Master Mix (SYBR Premix EX Taq^TM^, Dalian, China) on an ABI7500 qRT-PCR instrument (ABI, MA, USA), and the primers used are listed in Table S1.

### Yeast one-hybrid assays and electrophoretic mobility shift assays (EMSAs)

Yeast one-hybrid assays were conducted using a Matchmaker^TM^ Gold Yeast One-Hybrid Library Screening System (Clontech, Mountain View, CA, USA). The sequence including the underlined MBS element (TACCCTCTCATGTCCCTGTGAACCTAACGTAAGGCATTACGATTTGTAT) from the promoter of *VvACS1* was synthesized and inserted into a pHis2 vector. The ORF of *VvMYB14* was subsequently amplified and inserted into a pGADT7 vector. The resultant plasmid was introduced into the yeast strain Y1HGold. The detailed procedure was performed according to the user manual for this system, and 3-AT was used as a screening marker^54^.

For the EMSA experiment, the VvMYB14-His recombinant protein was obtained using a pEASY-E1 expression vector (TransGen Biotech, Beijing, China) and purified using His-tagged BeaverBeads™ IDA-Nickel (Beaver, BioBay, China). Oligonucleotide probes containing an MBS element (CATGTCCCTGTGAACCTAACGTAAGGCA) and a mutant probe (CATGTCCCTGTGTACATATCGTAAGGCA) were synthesized and labeled with biotin (Sangon, Shanghai, China). EMSAs were performed as described in the instruction manual included with the EMSA kit (Thermo Fisher Scientific, MA, USA) used. All the primers used are listed in Table S1.

### Transient cotransformation in tobacco leaves

The *VvMYB14* ORF was cloned and ligated into a pRI101-AN vector (Takara, Dalian, China) downstream of the *35* *S* promoter, yielding a *35* *S*::*MYB14* plasmid. The *VvACS1* promoter, the region up to 1500 bp upstream of ATG, and its mutated form with the mutant MBS element were used to replace the *35* *S* promoter within pRI101-GUS, yielding a *Pacs1*::*Gus* plasmid and a mutant *Pacs1*::*Gus* plasmid. The plasmids were subsequently introduced into *Agrobacterium* strain GV3101. The *Agrobacterium*-mediated transient transformation of tobacco leaves was performed according to the methods of Yang et al^55^. GUS histochemical staining and activity detection were then performed according to the methods of Jefferson et al.^56^.

### Transformation of *VvMYB14* into Merlot grape calli

The abovementioned *35* *S*::*MYB14* construct was used for sense overexpression. The 3′-UTR sequence of *VvMYB14* was cloned into a pRI101-AN vector for antisense suppression. The *VvMYB14* promoter fragments were isolated and inserted upstream of *35* *S* mini-*GUS*. The resultant constructs were introduced into *Agrobacterium* strain LBA4404, which was then transformed into grape calli according to a previous method^22^. The primers used in this experiment are listed in Table S1.

### Statistical analysis

Principal component analysis (PCA) was performed, and Pearson correlation coefficients (PCCs) were calculated using the statistical function prcomp and the cor function of R software (base package, v3.5.0). Orthogonal projections to latent structures-discriminant analysis (OPLS-DA) and variable importance in projection (VIP) values were generated using the R package MetaboAnlystR. Fisher’s exact test was applied to identify the significant KEGG pathways that had a false discovery rate (FDR) < 0.05. Analysis of variance (ANOVA) and significant difference tests were performed using SPSS (v19.0) software.

## Supplementary information

Table S12 DAMs arranged according to their distance to center dot in Y loading of O2PLS model

Fig. S1 Pearson’s correlation coefficients of FPKM values between pairs of samples

Fig. S2 In silico analysis of MYB binding site (MBS) in the promoter of VvACS1

Fig. S3 Expression responses of VvACS1 andVvMYB86 to MT (A) and yeast one-hybrid assay

Table S1 Primers used in this study

Table S2 All of the metabolites detected in the control berry skins and those treated with MT alone or MT plus 1-MCP

Table S3 DAMs in the control vs MT comparisons

Table S4 DAMs in the control vs MT_1-MCP comparisons

Table S5 DAMs in the MT_1-MCP vs MT comparisons

Table S6 DEGs between the control berry skins and the MT-treated berry skins

Table S7 DEGs between the berry skins treated with MT_1-MCP or MT

Table S8 Clusters of DEGs according to their expression patterns in the three samples

Table S9 Comparison of the variations in metabolites and their corresponding genes whose Pearson’s correlation coefficients are > 0.8 in the MT_1-MCP vs MT comparison

Table S10 KEGG enrichment of the DAMs and DEGs in the MT_1-MCP vs MT comparison

Table S11 DEGs arranged according to their distance to the center dot in the X loading of the O2PLS model

## Data Availability

The full RNA-seq data have been submitted to the Sequence Read Archive (SRA) of the NCBI under BioSample accession PRJNA646043 (https://www.ncbi.nlm.nih.gov/sra).
