# Supplementary figures and images for: Melatonin alters the secondary metabolite profile of grape berry skin by promoting VvMYB14-mediated ethylene biosynthesis
Source: Hortic Res. 2021 Mar 1;8:43. doi: 10.1038/s41438-021-00478-2 (PMC7917092; doi:10.1038/s41438-021-00478-2)

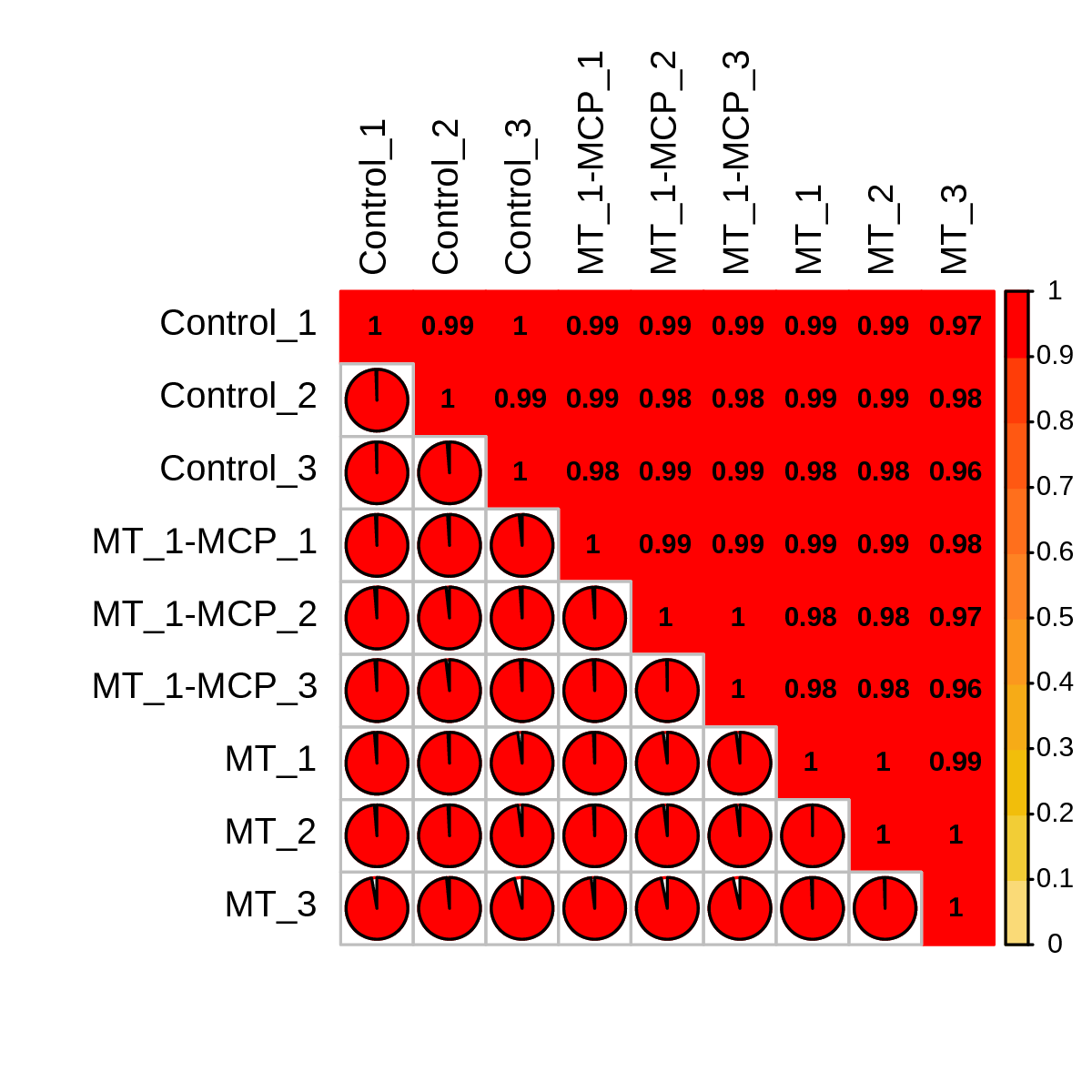


**Fig. S1** Pearson’s correlation coefficients of FPKM values between pairs of samples.

Supplement: Supplementary file 2 — Fig. S1 Pearson’s correlation coefficients of FPKM values between pairs of samples [file 41438_2021_478_MOESM2_ESM.docx]
