# Supplementary material for: Melatonin alters the secondary metabolite profile of grape berry skin by promoting VvMYB14-mediated ethylene biosynthesis
Source: Hortic Res. 2021 Mar 1;8:43. doi: 10.1038/s41438-021-00478-2 (PMC7917092; doi:10.1038/s41438-021-00478-2)
Supplement: Supplementary file 3 — Fig. S2 In silico analysis of MYB binding site (MBS) in the promoter of VvACS1 [file 41438_2021_478_MOESM3_ESM.docx]

AAGAGAGAAAAAAAAAAAGGTGAAAGAACGGTCTGGACTAATTTTTCTTTCCATGTATAGAAATCTCTCA -1547

CATCTTCATATGGTTTTGTTATTGAATCTTTTATTAACATTGAAGTGTTGGTTTTATATTCAAGAATCAA -1471

ATTGAATGATTGGTTCTATACTTATAAATCGATCACCCTCATAGTCCCGTGGTTGGACATTTGACCCATA -1401

AGCATGACTGTGATACTAATTTGTAATTATTTTCATTCCTTTCATATTCTTTAAAATCATTTGATGGAAT -1331

AACAATTGCGATAATCAGTTCTTTAAATCAAGTCAATCCTTTTACAAGTTTACACCCTTGGAATTGTACC -1261

CTCCATATAGTCTTGTTATCAAATCTTTTATTAACATTGAAGTGTTGGTTTAATATATATTCAAGAATTG -1191

AATAATTCATTCTATACACGAAGATCGATCACCCCTATAATCACATTGTTGGATAATTTGACTCATAAAC -1121

ATAACTTTTATACCAATTTGAAATTGTCTTCATTTATTTCACATTCTTTAAAATTATTTGGTGGAATAAT -1051

GATCGTGATAATCAGTCCACCAAAATAAGTCAATCCTGCTACAAGTCTACGACCTAGAATTGTCTCAATC -981

CTCTACTAACATTTTTTCCAATTTACACTACAATAATTCCCAAAAAAATGAGAATTGGGTCCATATTAAT -911

TTTAGATTTAACAGATGGAAACCTCTAATACCCACTGCGTAGTCTACTTGCCTGCTCGCTTTTTGGTACA -841

AAAGGAAACCATTGAGAACTTATTGGCTTAAAGATGGCCATCATTATATATTGAAAATCAAATGTAGGTT -771

GCGACATCTCACATCCGCCAACTAAAAGTGAAGTCGGTACCATGTACACTTATTTTTTTGATGACACTAC -701

CCCATTTGCTTTTGCCCCACCACAACCCTAACATTTTCCATAACCTGTCGGCCGATCTCCGTGTGCTGGC -631

CTTGGCTAACCGTCCTCCGACGGGGGTCCTCCCGACAATGAATATTTGCCGTGAACATTTTGTTATACTT -561

TATCTTATTTCTTCGTATTTTGGTCTGATATTTGAACTTTGATGGATACGAGTTTATATTGTTGTCTCCG -491

ATTAAATTTGTGTCTAGTGTTTTGTGTGACTCTTACCCGGACCAAAAGACAAAGGCAAAAAAGTCAAAAG -421

AGCCGCCCAATTTCAAACCTTGTGAATCTCCTGATTCTTCGTGGGAACTTCTTCCTCGTATTTTCCCACT -351

AAAAAATTTTCCCTCTGCAAGAGCCTAACAAATTAGATTCAACCTGGGAAAAAAAAATAGAATTCAGTAG -281

TCTTTTGGTTCT**TACCCTCTCATGTCCCTGTGAACCTAACGTAAGGCATTACGATTTGTATC**CCCACGTC -211

ATATGGTCACTTCCCATTTTTCTCATTTTCTGGGGGATTTGGCTTACCTTCTTACTTTGAAAATTTTCCA -141

TTTCCCCATTTTTGGTCCGTGTTCAACTGTACAGAGGTCTATAAAATTCTCTTGCATTCTCACATATTCC -71

CCCACAGCTATCGGCATTTCCCACTCTCATTGCTTTGCTAGCTACATATCCAGAGATACACTTAGAAACC -1

**ATG**

**Fig. S2 *In silico* analysis of MYB binding site (MBS) in the promoter of *VvACS1*.**

The MBS, highlighted with yellow color, was analyzed through PlantCARE Search Tool (http://bioinformatics.psb.ugent.be/webtools/plantcare/html/).
