# Supplementary material for: Melatonin alters the secondary metabolite profile of grape berry skin by promoting VvMYB14-mediated ethylene biosynthesis
Source: Hortic Res. 2021 Mar 1;8:43. doi: 10.1038/s41438-021-00478-2 (PMC7917092; doi:10.1038/s41438-021-00478-2)
Supplement: Supplementary file 4 — Fig. S3 Expression responses of VvACS1 andVvMYB86 to MT (A) and yeast one-hybrid assay [file 41438_2021_478_MOESM4_ESM.docx]

**A**


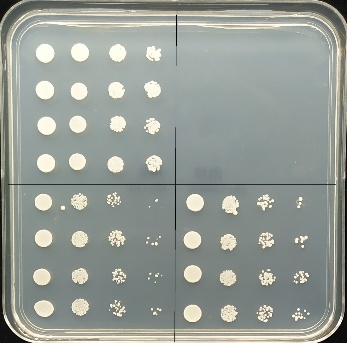


**B**

1

2

3

4

SD/-Try/-Leu/-His

100 mM 3-AT 0 mM 3-AT


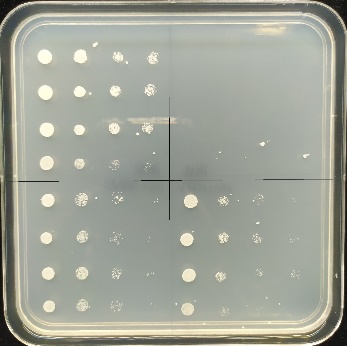


1

2

3

4

**Fig. S3 Expression responses of VvACS1 andVvMYB86 to MT (A) and yeast one-hybrid assay.** (1) P53-pHis2+pGADT7-REC2-53, Rec-P53 and the P53 promoter, whose interaction has been confirmed, acted as positive controls; (2) pHis2+pGADT7; (3) MBS-pHis2+pGADT7; (4) MBS-pHis2+pGADT7-MYB86. In panels 1-4, yeast cells were diluted 1, 10, 100 and 1000 folds from left to right. 3-AT (3-Amino-1,2,4-triazole) was used as a screening marker.
